# Maternal and neonatal outcomes among women with suspected macrosomia undergoing induction of labor: a systematic review and meta-analysis

**DOI:** 10.3389/fgwh.2026.1816012

**Published:** 2026-06-18

**Authors:** Hanan M. Al Kadri, Ashraf A. El-Metwally, Fathima F. Farook, Fatmah M. Othman, Hanan AlSehli, Atika A. Al Sudairy, Aljohrah I. Aldubikhi

**Affiliations:** 1 Department of Obstetrics and Gynecology, King Abdulaziz Medical City, Ministry of the National Guard-Health affairs, Riyadh, Saudi Arabia; 2 King Abdullah International Medical Research Center, Riyadh, Saudi Arabia; 3 College of Public Health and Health Informatics, King Saud bin Abdulaziz University for Health Sciences, Riyadh, Saudi Arabia; 4 College of Dentistry, King Saud Bin Abdulaziz University for Health Sciences, Riyadh, Saudi Arabia; 5 Department of Public Health, Saudi Electronic University, Riyadh, Saudi Arabia

**Keywords:** cesarean section, expectant management, induction of labor, macrosomia, meta-analysis, shoulder dystocia, systematic review

## Abstract

**Background:**

Fetal macrosomia, defined as birth weight ≥4,000 g, complicates 1%–10% of pregnancies and is associated with adverse maternal and neonatal outcomes including cesarean section, shoulder dystocia, and brachial plexus injury. The optimal management of suspected macrosomia induction of labor vs. expectant management remains controversial, with no standardized international guidelines.

**Objective:**

To comprehensively evaluate maternal and neonatal outcomes among women with suspected fetal macrosomia undergoing induction of labor compared with expectant management.

**Methods:**

We systematically searched MEDLINE, CINAHL, EMBASE, and the Cochrane Central Register of Controlled Trials from inception to December 2023. Randomized controlled trials and observational studies comparing induction of labor vs. expectant management in pregnancies with suspected fetal macrosomia were included. Primary outcomes were cesarean section, shoulder dystocia, Apgar score <7 at 5 min, and brachial plexus palsy. Pooled risk ratios with 95% confidence intervals were calculated using random-effects models. Subgroup analysis by study design and sensitivity analyses were performed. Risk of bias was assessed using the Cochrane tool for RCTs and the Newcastle-Ottawa Scale for observational studies.

**Results:**

Thirteen studies (4 RCTs, 9 observational; *N* = 4,442 women) were included. Induction of labor was not associated with a significant difference in cesarean section rates compared with expectant management (RR: 1.01; 95% CI: 0.83–1.23; *I*^2^ = 19%). No significant differences were observed for shoulder dystocia (RR: 0.99; 95% CI: 0.58–1.69; *I*^2^ = 23%) or brachial plexus palsy (RR 0.21; 95% CI 0.01–4.28). For Apgar score <7 at 5 min, conventional analysis showed no difference (RR: 0.92; 95% CI: 0.17–5.05; *I*^2^ = 78%); however, Peto's method for rare events demonstrated a significant reduction with induction (OR: 0.38; 95% CI: 0.25–0.57). Subgroup analysis revealed significant heterogeneity by study design for Apgar outcomes (*p* = 0.0003), with RCTs showing benefit (OR: 0.24; 95% CI: 0.15–0.39) and observational studies showing no effect. No publication bias was detected.

**Conclusion:**

Induction of labor for suspected fetal macrosomia does not significantly increase or decrease the risk of cesarean section or shoulder dystocia compared with expectant management. However, induction may improve neonatal outcomes as measured by Apgar scores, though this finding is driven by RCT evidence. The discrepancy between RCT and observational findings underscores the need for larger, well-designed trials to inform clinical guidelines. Clinicians should engage in shared decision-making, considering patient preferences and clinical context when managing suspected fetal macrosomia.

**Systematic Review Registration:**

https://osf.io/bkrc5.

## 1 Introduction

Macrosomia, defined as birth weight ≥ 4,000 g, is associated with substantial neonatal morbidity and mortality (1) and affects approximately 1%–10% of pregnancies (2, 3). Delivering a macrocosmic fetus poses high risk of various maternal complications including prolonged labor, emergency cesarean section, operative delivery, and postpartum hemorrhage. Fetal macrosomia is also associated with perinatal injuries, including shoulder dystocia, birth fractures, facial palsy, and brachial palsy (1, 4). The obstetric management of suspected macrosomia still controversial, therefore different practices have been applied (5, 6). To date, the literature suggests that planned cesarean section and induction of labor are treatment options for macrosomia to avoid adverse maternal and neonatal outcomes (6, 7). The American College of Obstetricians and Gynecologists states that elective cesarean section may be considered for non-diabetic women when the fetal weight is >5 kg (8). However, many studies indicated that performing elective cesarean section in case of suspected fetal macrosomia does not prevent neonatal injury (9–11). Other studies reported that early induction of labor can reduce the likelihood of fetal growth and subsequently reduce the associated complication of delivering macrocosmic fetus (11–13). However, this approach is the associated maternal and neonatal risk such as an increased risk of caesarean section deliver without reduction in birth related injuries (12).

Several studies reported that expectant management for women with suspected fetal macrosomia does not differ significantly from early induction in terms of incidence of cesarean section or perinatal injuries (13). Currently, knowledge about the appropriate management of macrosomia remains uncertain. The Cochrane review (14) provides high-quality evidence from RCTs, offering a strong foundation for understanding intervention effects. To build upon this work, our study augments the existing evidence by incorporating observational data, enabling a more comprehensive assessment of real world clinical outcomes.

Our study enhances the evidence base by incorporating observational studies to provide a more comprehensive understanding of clinical outcomes. This approach allows us to examine the generalizability of RCT findings to real world practice settings, analyze cesarean section rates and perinatal outcomes across different study designs, and evaluate additional clinically relevant outcomes such as Apgar scores that were not extensively examined in previous syntheses. By including more recent evidence and complementary study designs, our analysis aims to provide a more complete picture of management options for suspected fetal macrosomia.

Despite existing research, including trials suggesting that induction at 39 weeks may reduce cesarean sections (14), the optimal management of suspected macrosomia remains debated (15). The current lack of standardized policies or evidence-based guidelines regarding early induction vs. expectant management for fetal macrosomia (11) underscores the need for comprehensive evidence synthesis. This systematic review and meta-analysis therefore aims to comprehensively evaluate comparative outcomes between labor induction and expectant management in pregnancies with suspected fetal macrosomia. We conducted separate analyses for randomized controlled trials and observational studies to distinguish between high-quality experimental evidence and real-world observational data.

## 2 Material and methods

### 2.1 Search study and database

This systematic review and meta-analysis followed the Preferred Reporting Items for Systematic Reviews and Meta-analyses(PRISMA) guidelines (16). The review protocol was registered on OSF at https://osf.io/bkrc5. We searched MEDLINE, CINAHL, EMBASE, and the Cochrane Central Register of Controlled Trials (CENTRAL) from inception to December 2023, using combination of the following search terms: “induction of labor vs. expectant management”, “labor induction”, “expectant management”, “maternal outcomes”, “neonatal outcomes”, “C- section”, “shoulder dystocia”, “Apgar score at 5 min”, and “brachial/Erbs palsy”.

Additionally, we performed a manual search of reference lists from included studies and relevant reviews.

Titles and abstracts were screened to identify potentially eligible studies. The quality criteria of the individual articles were reviewed by two independent reviewers, and the results were compared. The third reviewer resolved all conflicts.

### 2.2 Inclusion and exclusion criteria

The studies that compared the expected management vs. early induction of labor among women with suspected fetal macrosomia were included in this review. Participants were pregnant women with fetus suspected macrosomia who had no other indication for early induction of labor. The outcomes that were extracted from those studies include (C-section, shoulder dystocia, Apgar score at 5 min, and brachial/Erb's palsy). We included randomized controlled trials (RCTs) and observational studies (including cohort, case-control, and analytical cross-sectional designs). Meanwhile, the studies were excluded if they had any of the following criteria: insufficient information related to study outcomes, Studies were excluded if they did not report relevant outcomes or provided insufficient data for analysis.

### 2.3 Data extraction

Data extraction was performed independently by two reviewers using a standardized Excel spreadsheet. Extracted data included author, publication year, country, sample size, number of events in the intervention and control groups, outcomes measured, and study design. Any discrepancies were resolved through discussion or consultation with a third reviewer.

Study selection was conducted in two stages. First, titles and abstracts were screened to identify potentially eligible studies. This was followed by full-text screening to confirm eligibility based on the predefined inclusion and exclusion criteria.

Extracted data were organized into a structured database. Outcome-specific data were collected for cesarean section, shoulder dystocia, Apgar score at 5 min, and brachial plexus (Erb's) palsy. For each outcome, the number of events and total sample size in both the intervention and control groups were recorded.

Risk of bias assessment was also conducted independently by two reviewers. The Cochrane Risk of Bias tool was used for randomized controlled trials, and the Newcastle–Ottawa Scale (*N*OS) was applied for observational studies (17, 18). Disagreements were resolved by consensus or by involving a third reviewer.

For the RCT, the assessment tool has seven domains, assessing random sequence generation, allocation sequence concealment, blinding of participants and personnel, blinding of outcome assessment, completeness of outcome data, selective reporting, and other sources of bias. Based on the assessment, the quality ranged from low risk, high risk, or unclear risk.

### 2.4 Statistical analysis

Data on the number of events and total participants in both groups were used for each outcome. Study weights were assigned using the inverse variance method. A random-effects model was applied to estimate pooled effect sizes, accounting for both within-study and between-study variability, given the anticipated clinical and methodological heterogeneity across studies.

Statistical heterogeneity was assessed using the I² statistic and the chi-square test, with corresponding degrees of freedom and *p*-values reported (19, 20). Funnel plots were constructed to assess potential publication bias by examining the relationship between effect estimates and their standard errors. Visual inspection of funnel plot symmetry, along with Egger's regression asymmetry test, was used to evaluate small-study effects (21). All statistical analysis has been performed using R.

#### 2.4.1 Subgroup analysis

To explore potential sources of heterogeneity, subgroup analyses were performed based on study design (randomized controlled trials vs. observational studies). This approach allows differentiation between evidence derived from experimental settings and real-world observational data.

#### 2.4.2 Sensitivity analysis

Sensitivity analyses were conducted to assess the robustness of the findings by excluding studies with a high risk of bias.

## 3 Results

### 3.1 Study selection process/eligible studies

**Figure 1:**
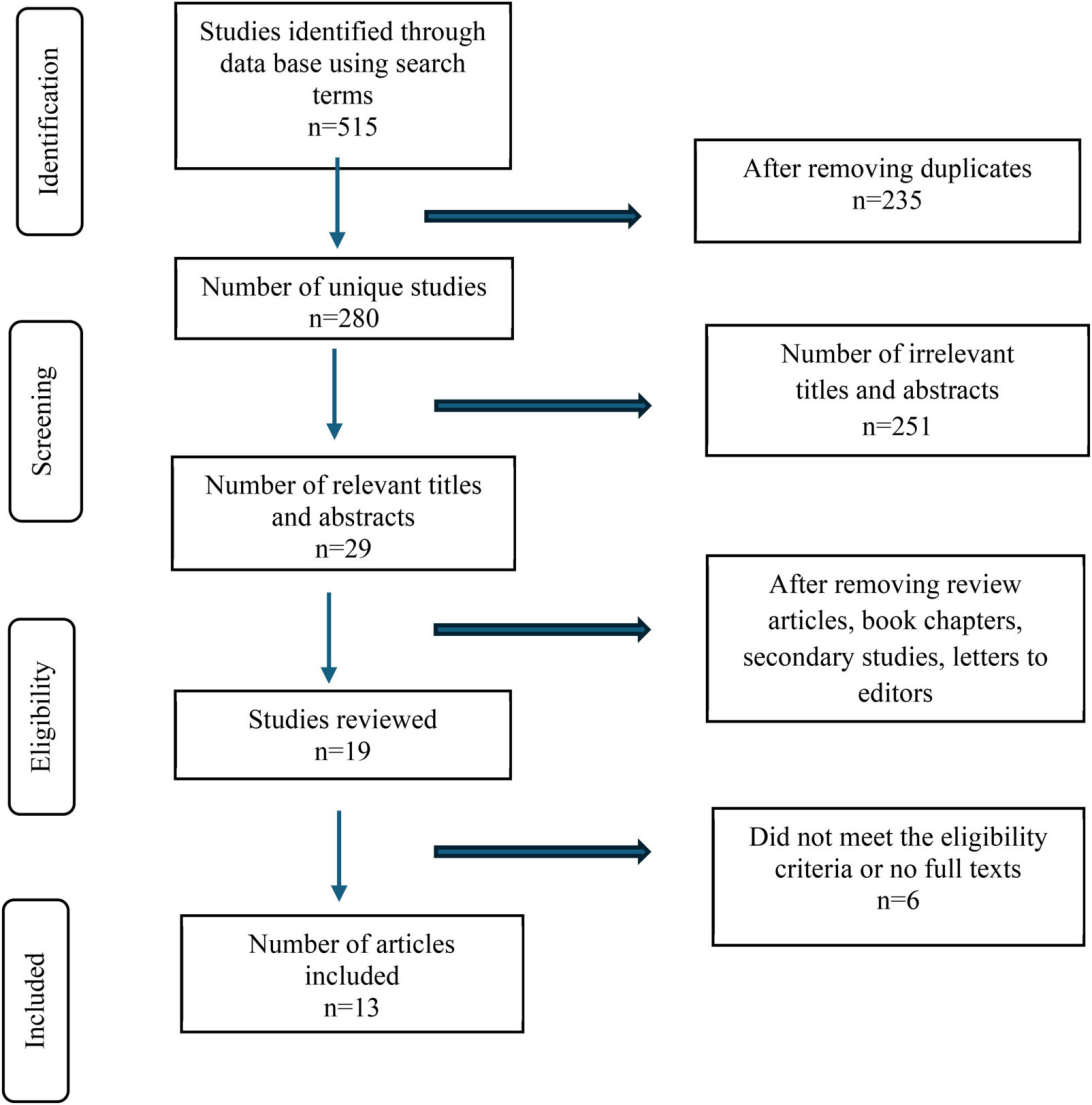
PRISMA flowchart summarizing inclusion in meta-analysis of relevant citations and final number of included studies.

In total, 515 studies were retrieved through databases searching. We excluded 235 duplicated articles and 251 records that were irrelevant to the selection criteria. After removal of duplicates and title/abstract screening, 19 studies underwent full text screening, after which the full set of eligibility criteria was applied. After full text evaluation, 13 studies remained eligible for inclusion in this review, as outlined in Figure 1 based on PRISMA (Preferred Reporting Items for Systematic Reviews and Meta-Analyses) guidelines.

#### 3.1.1 Characteristics of the eligible studies

Table 1 shows the characteristics of the included studies. The included studies were conducted in different countries, mainly high-income countries, of which five were conducted in the USA (9, 22–25) two in Denmark (26, 27), one each in Israel (28), Italy (29), Norway (30), Germany (31) and the United Kingdom (32), and one multicenter study in France, Switzerland and Belgium (33). As shown in Table 1, 9 studies were observational and 4 were RCTs. All included studies were conducted on pregnant women in labor, and the total sample size of the studies ranged from 40 to 838.

**Table 1:** Summary of characteristics of 13 included studies comparing maternal and neonatal outcomes of women experienced with induction labor compared to women with expectant management (*n* = 13).

Author	Year	Country	Study design	Sample size	Outcome studied	Risk of Bias*
Bars et al. (31)	1984	Germany	Observational	556	C-Section,	High
Johansen et al. (26)	1987	Denmark	Observational	477	C-Section, Apgar score <5 at 7 min	Medium
Larsen et al. (19)	1991	Denmark	Observational	838	C-Section, Shoulder dystocia, Apgar score <5 at 7 min	High
Delpapa et al. (9)	1991	USA	Observational	162	C-Section	Low
Combs et al. (23)	1993	USA	Observational	159	C-Section, Shoulder dystocia,	Medium
Friesen et al. (22)	1995	USA	Observational	186	Shoulder dystocia, Apgar score <5 at 7 min	Medium
Tey et al. (24)	1995	USA	RCT	40	C-Section, Shoulder dystocia, Apgar score <5 at 7 min, and Brachial palsy	Low risk of bias
Diani et al. (29)	1995	Italy	Observational	457	C-Section	Low
Gonen et al. (28)	1997	Israel	RCT	273	C-Section, Shoulder dystocia, and Brachial palsy	High risk of bias
Leaphart et al. (25)	1997	USA	Observational	106	C-Section, Shoulder dystocia	Low
Thornton, (32)	1998	UK	RCT	59	C-Section, Shoulder dystocia, and Brachial palsy	low risk of bias
Wojtasinka et al. (30)	2000	Norway	Observational		C-Section, Shoulder dystocia, Apgar score <5 at 7 min	Medium
Boulvain et al. (33)	2015	Multicounty	RCT	818	C-Section, Shoulder dystocia, Apgar score <5 at 7 min, and Brachial palsy	Unclear risk of bias

* Risk of bias was assessed by the Cochrane Risk of Bias Tool for RCTs and the Newcastle-Ottawa Scale for observational studies.

### 3.2 Assessment of the risk of bias in the included studies

The results of the quality assessment of the studies are provided in Table 1. For RCTs, one has low risk of bias (24), two had unclear risk of bias (32, 33), while one has high risk of bias (28) due to deviations from the intended intervention. The majority of the observational studies had a moderate risk of bias.

#### 3.2.1 Pooled risk ratio for cesarean section

**Figure 2:**
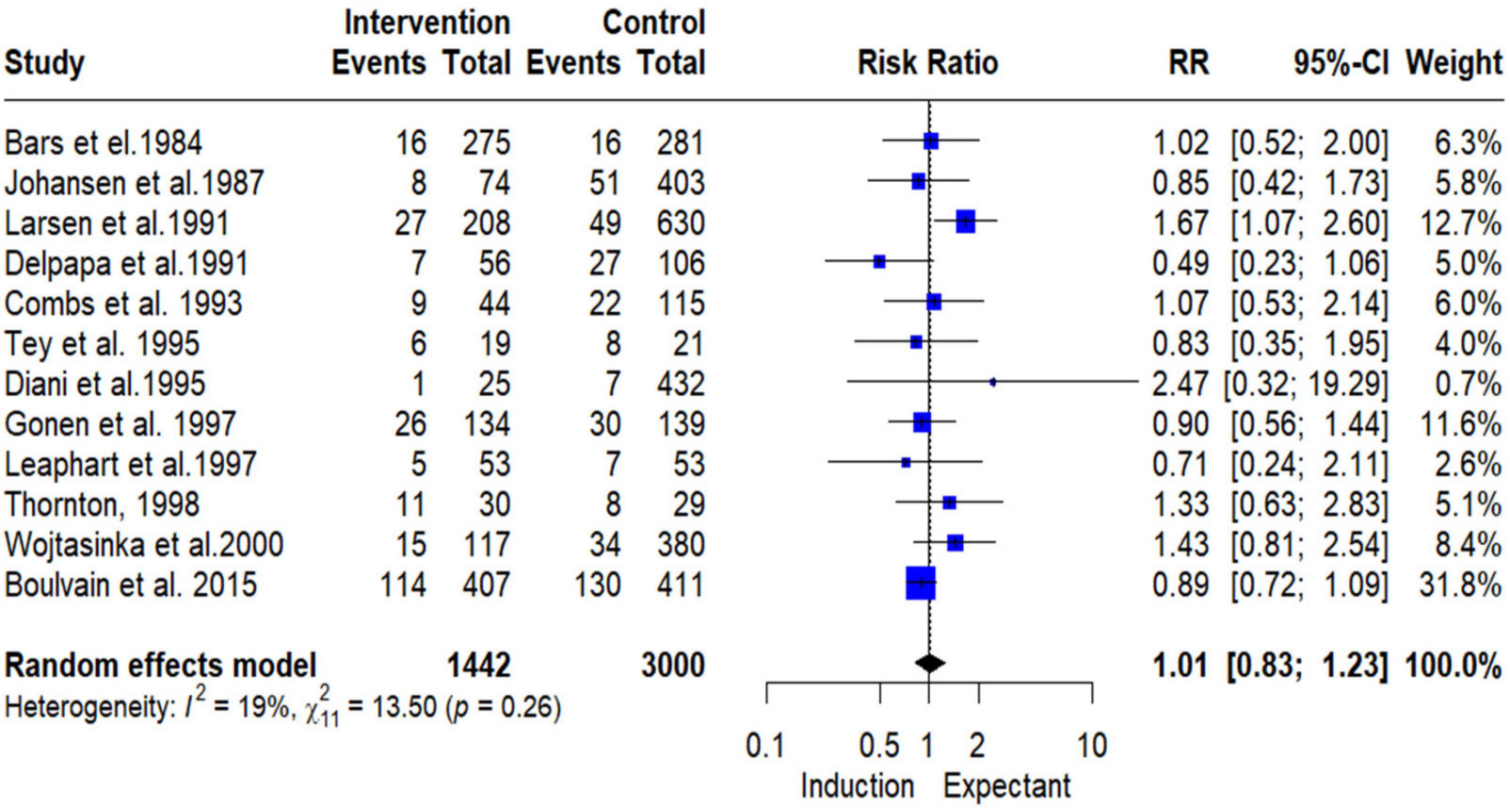
Forest plot depicting the difference in the *risk of C-Section* between Labor induction and Expectant management of Labor **(*n*** **=** **12)**.

Figure 2 below shows the forest plot depicting the difference in risk of cesarean section between labor induction and expectant management of labor. The number of studies contributing to cesarean section totaled 12 studies, conducted between 1984 and 2015, with a sample size of 4,442 women suspected fetal macrosomia included in the meta-analysis. Almost 100% of these studies were from high-income countries. Women who had induction of labor had similar risk of cesarean delivery compared with those who had expectant management for suspected fetal macrosomia (RR: 1.01; 95% Cis: 0.83–1.23), Figure 2. Statistical heterogeneity within the studies was low (*I*^2^ = 19%, *X*^2^:13.50), with the *p*-value = 0.26).

#### 3.2.2 Subgroup analysisfor cesarean section by study design

Subgroup analysis was performed to assess whether study design influenced the association between labor induction and cesarean section. Among observational studies (8 studies; 3,252 women), induction of labor was associated with a slightly higher but non-significant risk of cesarean delivery compared with expectant management (RR = 1.08; 95% CI: 0.80–1.47), with moderate heterogeneity (*I*² = 31.1%). In randomized controlled trials (4 studies; 1,190 women), induction of labor showed a non-significant trend toward a lower risk of cesarean section (RR = 0.91; 95% CI: 0.76–1.09), with no observed heterogeneity (*I*² = 0%). There was no statistically significant difference between subgroups based on study design (p for subgroup differences = 0.32).

#### 3.2.3 Pooled risk ratio for shoulder dystocia

**Figure 3:**
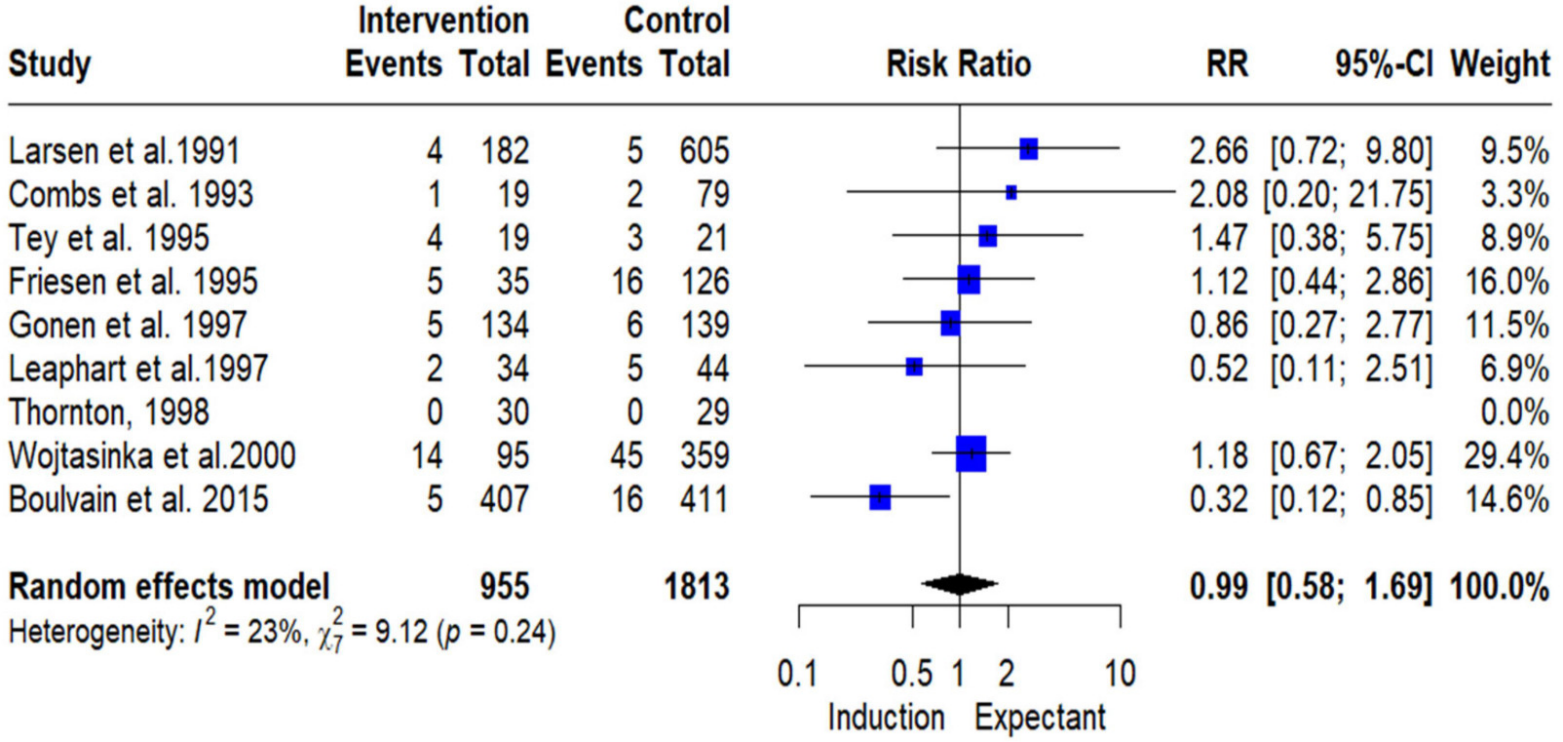
Forest plot depicting the difference in the risk of shoulder dystocia between labor induction and Expectant management of labor (*n* = 8).

The forest plot comparing the risk of shoulder dystocia is presented in Figure 3. The number of studies that contributed to the risk of shoulder dystocia totaled 8 studies conducted between 1991 and 2015, with a sample size of 2,768, with all studies conducted in high-income countries. There were no statistically significant differences in risk of shoulder dystocia between labor induction and expectant management of labor (RR: 0.99; 95% Cis: 0.58–1.69, *p* = 0.24, *I*^2^ = 23%).

Subgroup analysis by study design demonstrated no statistically significant difference in the risk of shoulder dystocia between labor induction and expectant management in observational studies (RR = 1.22; 95% CI: 0.80–1.86) or randomized controlled trials (RR = 0.68; 95% CI: 0.27–1.68). There was no evidence of a subgroup effect based on study design (p for subgroup differences = 0.25).

#### 3.2.4 Pooled risk ratio for apgar score less than 7 at 5 min

**Figure 4:**
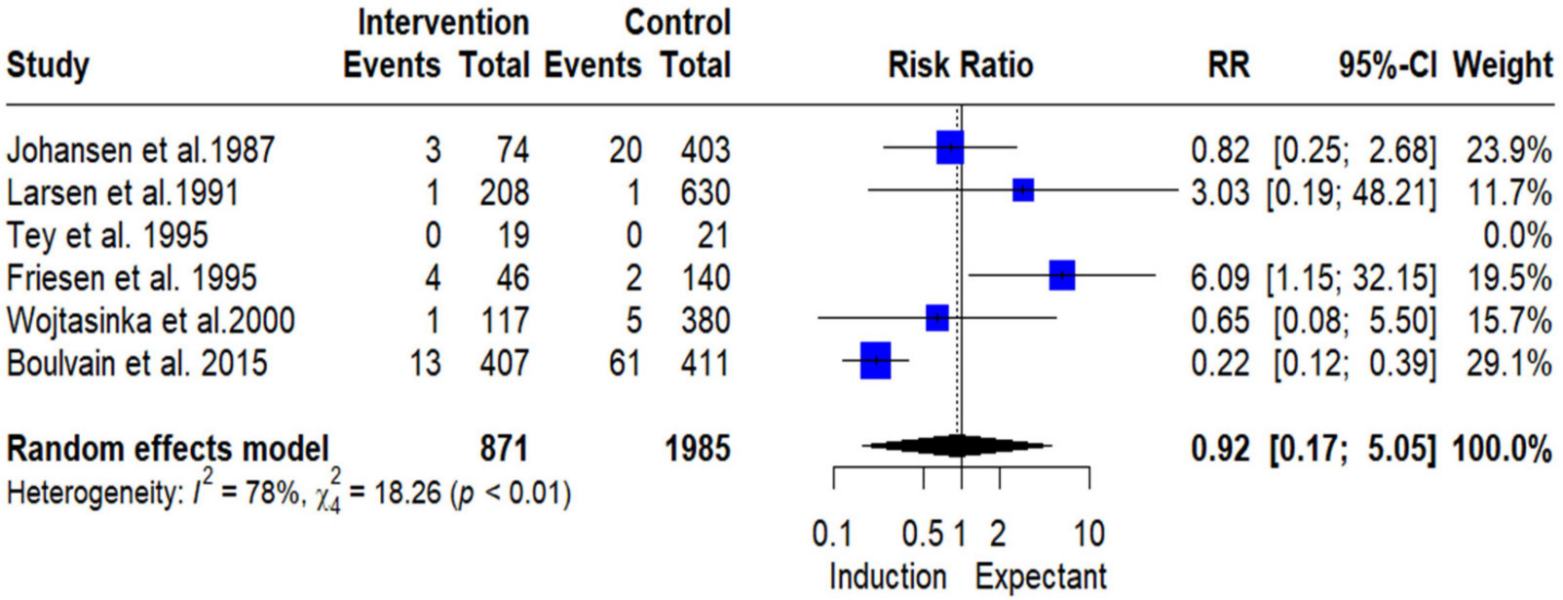
Forest plot depicting the difference in the *risk of Apgar score < 7 at 5 min* between labor induction and Expectant management of labor (*n* = 6).

The point estimate for the difference in the risk of Apgar score <7 at 5 min between labor induction and expectant management of labor is shown in Figure 4. The number of studies that contributed to the risk of shoulder dystocia totaled 5, conducted between 1987 and 2015, with a sample size of 2,856, with all studies conducted in high-income countries. The results showed that there was no difference in the Apgar score at 5 min among women who had delivered by labor induction compared to management in anticipation of delivery (RR: 0.92; 95% Cis: 0.17–5.05, *p* ≤ 0.01, *I*^2^ = 78%).

Using Peto's method for odds ratios (recommended for rare events), induction of labor significantly reduced the overall risk of Apgar scores <7 at 5 min (OR: 0.38, 95% CI: 0.25–0.57, *p* < 0.0001). Subgroup analysis revealed significant differences between study designs (P for interaction = 0.0003). RCTs demonstrated a clear benefit (OR: 0.24, 95% CI: 0.15–0.39), while observational studies showed a non-significant trend toward increased risk (OR: 1.46, 95% CI: 0.63–3.39).

#### 3.2.5 Brachial

The number of studies that contributed to the risk of brachial palsy totaled 4 studies conducted between 1995 and 2015, with a sample size of 1,190, with all studies conducted in high-income countries. The results showed that there was no difference in the risk of brachial palsy in newborns whose mothers had delivered by induction of labor compared to pregnancy management (RR: 0.21; 95% Cis: (0.01, 4.28). However, only one study by Gonen et al. (28) contributed to the risk ratio, as no events occurred in either the intervention or control group in the other 3 studies. Therefore, a forest plot could not be generated.

#### 3.2.6 Publication bias

**Figure 5:**
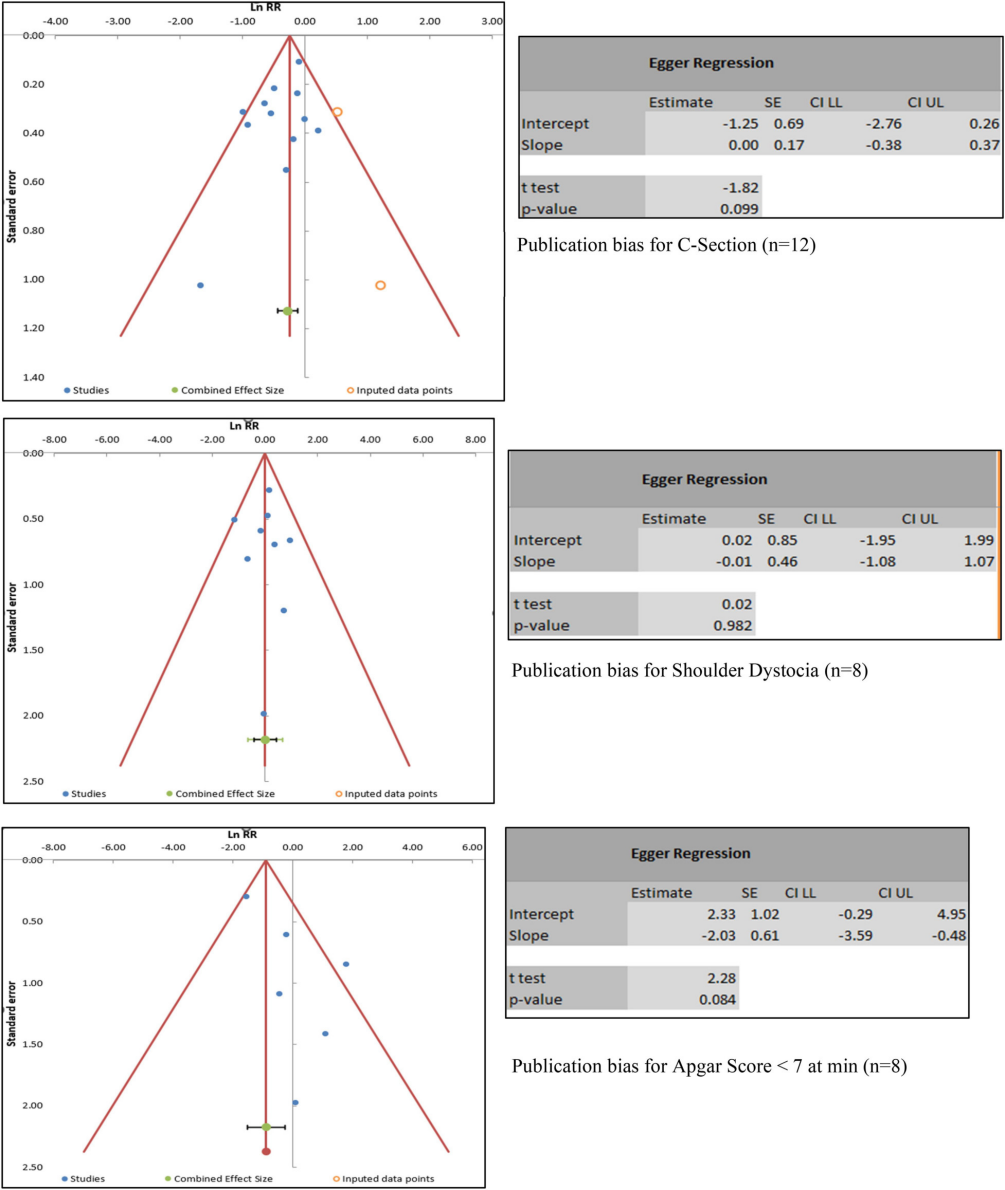
Funnel plot depicting publication bias.

There was no publication bias for other outcomes such as cesarean section, shoulder dystocia and Apgar score for <7 at 5 min, as shown by the symmetrical shape of the funnel plots (Figure 4). The evidence of the funnel plot was confirmed with a quantitative method by Egger's regression test, a linear regression test for the asymmetry of the funnel plot. The results of the Egger's regression test showed that the intercept of the regression model was less than and the *p*-value > 0.05, indicating that there was no publication bias (Figure 5).

## 4 Discussion

This meta-analysis investigated the effect of induction of labor as a management strategy in pregnancies with suspected fetal macrosomia to prevent maternal and perinatal complications, including cesarean section, shoulder dystocia, Apgar score <7 at 5 min, and brachial plexus injury. It is important to emphasize that “suspected macrosomia” in the included studies is a clinical surrogate based primarily on estimated fetal weight and does not account for fetal body proportionality or cephalopelvic dynamics, which are highly relevant to the feasibility of vaginal delivery. In addition, antenatal estimation of fetal weight has limited accuracy, with a considerable proportion of fetuses being both overestimated and underestimated. This misclassification may attenuate true effect estimates, as many included pregnancies may not have met actual birthweight criteria for macrosomia. Therefore, this review evaluates management strategies applied to pregnancies labeled as macrosomic rather than the intrinsic biomechanical suitability of vaginal birth for large fetuses. Overall, the results showed that induction of labor is not associated with significant differences in adverse maternal or perinatal outcomes.

For this meta-analysis, majority of the study were observational studies rather than RCTs. Our findings align with the Cochrane review by Boulvain et al. in 2023 (14), which included four RCTs and found no significant difference in cesarean section rates between induction and expectant management for suspected macrosomia. However, the Cochrane review reported a reduction in shoulder dystocia and fractures, outcomes our meta-analysis did not replicate, possibly due to the inclusion of observational studies with potential confounding. While the Cochrane review focused exclusively on RCTs, our study incorporated both RCTs and observational data to provide a broader perspective, albeit with limitations in internal validity. The findings of the current study are also consistent with prior RCT evidence indicating that induction of labor is not associated with a reduction in cesarean delivery rates (34).

A key finding of this review is the statistically significant divergence between randomized controlled trials and observational studies, suggesting that residual confounding and selection bias in observational studies may have influenced pooled effect estimates, particularly for cesarean section and neonatal outcomes such as Apgar scores.

Similarly, a Cochrane review reported no significant effect of induction of labor on cesarean section risk (35). The use of Peto's method, which appropriately handles studies with zero events, revealed a significant overall benefit of induction that was not apparent in conventional analyses. This highlights the sensitivity of rare event outcomes to the choice of statistical model and suggests that conventional random-effects models may underestimate treatment effects when event rates are low. The persistent divergence between RCT and observational findings (*p* = 0.0003) further supports the likelihood that unmeasured confounding and selection bias in observational studies may obscure true treatment effects.

In addition, the authors of the Cochrane review found no significant difference in APGAR score <7 at 5 min and brachial plexus injury between women undergoing induction of labor and expectant management, reflecting the consistency of the results with our meta-analysis (35). In that review, gestational age was determined at or before term (37 to 40 weeks). In cases of fetal macrosomia, it is recommended that labor be induced either at term or near term. However, the exact weeks of gestation were not specified. Some guidelines recommend considering induction of labor (IOL) at 38 + 0 weeks gestation if the estimated fetal weight is 3,500 g at 36 weeks, 3,700 g at 37 weeks, or 3,900 g at 38 weeks (36). Variability in both the definition of suspected macrosomia and the gestational age at which induction was undertaken likely contributed to clinical heterogeneity across studies. Such variability may influence neonatal outcomes, as earlier or later induction thresholds could affect fetal maturity and birthweight at delivery, thereby reducing comparability across studies.

Furthermore, our results are consistent with a prior review by Sanchez-Romas et al. (13), in which the authors found no difference in the incidence of shoulder dystocia between the two management protocols (13). On the other hand, the finding of the current study is not in line with prior systematic review of C-sections conducted more than two decades earlier, which used observational studies to determine the benefits of expectant management over induction of labor (13). Since the conclusion of the superiority of expectant management over induction of labor is mainly based on observational studies, it is possible that the results are biased due to the problem of unmeasured confounding factors in observational studies (37, 38).

In contrast to RCTs, in which women are randomly assigned to the two management options (39), physicians in the observational studies may have assigned women to either expectant management or induction of labor depending on their risk profile. Thus, women for whom induction of labor is indicated may have different indications for cesarean section than women for whom expectant management is indicated. Moreover, detailed indications for cesarean section (e.g., non-progress of labor vs. suspected fetal distress) were inconsistently reported across the included studies, limiting the ability to determine whether differences in cesarean rates were driven by labor-related factors or fetal compromise. It is also possible that macrosomia was not the only reason for induction of labor in some women (40). One can speculate that obstetricians who tend to lean towards labor induction are also more likely to perform cesarean sections. It is likely that the results of the observational studies in the review by Sanchez-Romas et al. favor the expectant management (13). If we summarize these observational studies, the beneficial effect of expectant management is weakened. Since the biases cannot be avoided in the quantitative synthesis due to the selection of study participants and unmeasured confounding factors, the results of the prior review by Sanchez-Romas et al. (13) must be interpreted in light of the limitations of observational studies (13).

### 4.1 Strengths and limitations

This meta-analysis combined the results of both observational and RCTs that have been published on this topic so far. The symmetrical shape of the forest plots suggests that there is no publication bias, which means that our study results are not influenced by small study effects. However, the finding of the current study need to be interpreted with some cautions. First, while our study includes both RCTs and observational data, the latter introduces potential biases such as unmeasured confounding. The Cochrane review (14) avoided this limitation by focusing solely on RCTs but was constrained by the small number of trials. Our inclusion of observational studies provides a broader but less rigorous perspective, highlighting the need for further RCTs to resolve discrepancies.

In addition, although the included studies were multi-country in nature, most were from high-income countries, so we may not be able to draw firm conclusions to generalize the results to low-income countries. Adding to this, the definition of macrosomia was not uniformly defined across the studies as well as the variation in the gestational age where the induction was suggested.

## 5 Conclusion

Our study found no significant differences in maternal or neonatal outcomes between induction and expectant management for suspected macrosomia. While these results contrast with the Cochrane review's findings of reduced shoulder dystocia and fractures, the discrepancy may stem from our inclusion of observational studies. Future research should prioritize large, well-designed RCTs to clarify these outcomes and refine gestational age thresholds for induction.

## Data Availability

Publicly available datasets were analyzed in this study. This data can be found here: The datasets generated for this systematic review and meta-analysis (including search strategies, extraction forms, and analysis code) are available in the Open Science Framework repository: https://osf.io/bkrc5.
